# Brite Adipocyte FGF21 Attenuates Cardiac Ischemia/Reperfusion Injury in Rat Hearts by Modulating NRF2

**DOI:** 10.3390/cells11030567

**Published:** 2022-02-06

**Authors:** Hanbyeol Moon, Jung-Won Choi, Byeong-Wook Song, Il-Kwon Kim, Soyeon Lim, Seahyoung Lee, Gyoonhee Han, Ki-Chul Hwang, Sang Woo Kim

**Affiliations:** 1 Department of Integrated Omics for Biomedical Sciences, Graduate School, Yonsei University, Seoul 03722, Korea; moonstar3636@yonsei.ac.kr (H.M.); gyoonhee@yonsei.ac.kr (G.H.); 2 Institute for Bio-Medical Convergence, College of Medicine, Catholic Kwandong University, Gangneung-si 25601, Korea; gardinia@hanmail.net (J.-W.C.); songbw@ish.ac.kr (B.-W.S.); ilkwonkim@cku.ac.kr (I.-K.K.); slim724@cku.ac.kr (S.L.); sam1017@ish.ac.kr (S.L.); kchwang@cku.ac.kr (K.-C.H.); 3 Catholic Kwandong University, International St. Mary’s Hospital, Incheon Metropolitan City 22711, Korea

**Keywords:** brite adipocyte, cardiac ischemia/reperfusion, cardioprotection, FGF21, NRF2

## Abstract

Although the optimal therapy for myocardial infarction includes reperfusion to restore blood flow to the ischemic area, myocardial injury after ischemia/reperfusion usually leads to an inflammatory response, oxidative stress, and cardiomyocyte apoptosis. In this study, rat adipose-derived stem cells were differentiated into low-thermogenic beige adipocytes (LBACs) and high-thermogenic beige adipocytes (HBACs) to study the different cardioprotective effects of heterogeneous expression of brown adipocytes. We found that antioxidant and antiapoptotic factors in H9c2 cardiomyocytes were upregulated by high levels of secreted FGF21 in HBAC conditioned medium (HBAC-CM), whereas FGF21 in HBAC-CM did not affect antioxidative or antiapoptotic cell death in H9c2 cardiomyocytes with *Nrf2* knockdown. These results show that NRF2 mediates antioxidative and antiapoptotic effects through the HBAC-secreted factor FGF21. Consistent with this finding, the expression of antioxidant and antiapoptotic genes was upregulated by highly secreted FGF21 after HBAC-CM treatment compared to LBAC-CM treatment in H9c2 cardiomyocytes via NRF2 activation. Furthermore, HBAC-CM significantly attenuated ischemic rat heart tissue injury via NRF2 activation. Based on these findings, we propose that HBAC-CM exerts beneficial effects in rat cardiac ischemia/reperfusion injury by modulating NRF2 and has potential as a promising therapeutic agent for myocardial infarction.

## 1. Introduction

Ischemic heart disease including acute myocardial infarction is the leading cause of morbidity and mortality, and appropriate restoration of blood flow (reperfusion) to the ischemic myocardium is indispensable [1,2]. However, this reperfusion may cause further myocardial ischemia/reperfusion (IR) injury that can induce heart failure and endothelial and microvascular dysfunction and have more serious adverse effects on other myocardial tissues. Oxidative stress is one of the most critical mechanisms of the complicated networks associated with cardiovascular diseases such as myocardial IR injury [3,4]. Oxidative stress mediated by bursts of reactive oxygen species (ROS), which play critical roles in cardiovascular diseases, exacerbate cellular processes that promote cell death and cardiac dysfunction [5,6]. Since the development of oxidative stress after reperfusion and its effects on damage are associated with various mechanisms, controlling specific pathways can be more therapeutically advantageous than administering antioxidants. Among several antioxidant defense systems, nuclear factor (erythroid-derived 2)-like 2 (NRF2), a nuclear transcription factor, exerts essential regulatory effects on defense genes encoding detoxification enzymes and antioxidant proteins. NRF2 thereby participates in cellular resistance to oxidative agents and induces the responses of endogenous antioxidant enzymes [7]. Therefore, the regulation of antioxidative stress by NRF2 could be a critical target in reducing the risk of IR injury [8].

*Nrf2* has a basic leucine zipper (bZip) domain that regulates its downstream antioxidant gene expression in response to oxidative stress and is a member of the Cap ‘n’ Collar (CNC) family of transcription factors [9]. Under normal conditions, NRF2 binds to Kelch-like ECH-associated protein 1 (KEAP1) and is located in the cytoplasm. KEAP1 prevents nuclear entry and facilitates ubiquitination and degradation of NRF2. However, when exposed to oxidants, NRF2 is separated from KEAP1 and moves to the nucleus. There, NRF2 binds with antioxidant response elements (AREs) to promote the expression of antioxidant genes such as glutathione reductase (GR), heme oxygenase-1 (HO-1), NAD(P)H:quinone oxidoreductase 1 (NQO1), Catalase, sestrin2 (SESN2), and superoxide dismutase 1 (SOD1) [9,10,11]. The NRF2/HO-1 signaling pathway is implicated in the defense against various oxidative stress inducers and may be a promising target for IR inhibition. Many drugs or extracts such as resveratrol and triptolide have been reported to inhibit oxidative stress by activating NRF2/HO-1 signaling to limit infarct size and improve cardiac function. Signaling molecules upstream of NFR2 such as phosphatidylinositol-3-kinase (PI3K), protein kinase C (PKC), silent information regulator 1 (SIRT1), and glycogen synthase kinase 3 (GSK3), which promote degradation and inactivation of NRF2, are also targets for treatment.

Adipose tissue (AT) contains pluripotent adipose-derived stromal cells (ASCs) with the same differentiation and regenerative potential as bone marrow-derived mesenchymal stromal cells (MSCs) [12]. Therefore, infusion of higher concentrations of ASCs may have a stronger therapeutic effect in patients with ischemic heart disease, and ASCs have been reported to exhibit greater angiogenesis than MSCs, potentially increasing myocardial perfusion and regeneration in chronic ischemic heart disease [12,13]. Furthermore, clinical care with ASCs primed against endothelial cell lineages can potentially induce angiogenesis because in vitro, a serum-free environment and vascular endothelial growth factor (VEGF) stimulation promote differentiation of MSCs and ASCs into endothelial cell lineages [14]. These differences between ASCs and MSCs are thought to be caused by differences in cell origin, cell population, and cell structure. Since the ASCs can differentiate into multiple lineages and release various cytokines associated with immunomodulation, they may have an eminent role in tissue regeneration [12]. Recently, it has been reported that factors secreted by AT such as adiponectin may control cardiac pathology or interact with neurohormones in heart failure (HF) with preserved ejection fraction (HFpEF) [15,16]. Similarly, cardiokines released from cardiac cells due to environmental changes play pathological roles in distant tissues such as muscle, fat, and liver tissues and as regulators of apoptosis, growth, fibrosis, and remodeling [17,18]. However, much less is known about their regulatory roles in metabolic interactions between the heart and other organs. These findings suggest the existence of communication between the heart and the surrounding tissues.

Fibroblast growth factor 21 (FGF21) is an endocrine regulator with many metabolic effects on processes including weight loss, glucose and lipid metabolism, and insulin sensitivity [19]. It has been reported that FGF21 can reduce organ injury through diverse mechanisms including an antioxidative effect through the upregulation of NRF2 [20,21,22,23]. Previous studies have shown that FGF21 induces cardioprotection from IR injury by inhibiting oxidative stress and apoptosis via increases in antioxidant gene and enzyme expression [24]. Although FGF21 is known to be produced mainly by the liver, recent studies have shown that BAT also secretes FGF21 [25,26]. However, it is still unknown whether BAC-derived FGF21 plays an endocrine role in oxidative stress in the context of IR injury.

We investigated whether white adipocytes differentiated from human ASCs (hASCs) could increase brown adipogenesis by expressing uncoupling protein 1 (UCP-1) and PR/SET domain 16 (PRDM16) due to small molecule treatments in a previous study [27]. In this study, we demonstrated that the activity of NRF2 was increased by FGF21, a factor secreted from beige adipocytes (BACs), which leads to increases in antioxidative effects and subsequently reduces apoptosis, thus alleviating IR injury. These findings demonstrate that FGF21 release from BACs exerts an endocrine protective effect against IR injury. The aim of this study was to test the conditioned media of brown adipocytes, which participates in the regulation of antioxidant and antiapoptotic response in IR rat hearts.

## 2. Materials and Methods

### 2.1. Animal Experiments

All animal experiments were approved by the Committee for the Care and Use of Laboratory Animals of the College of Medicine of Catholic Kwandong University (CKU 01-2019-008). An IR injury model was induced in male Sprague-Dawley rats at eight weeks (*n* = 10 per group). The rats underwent 60 min of ischemia followed by 48 h of reperfusion. The animals were given oxygen by a ventilator (Harvard Apparatus, Holliston, MA, USA) and then subjected to median sternotomy. The left anterior descending coronary artery (LAD) was blocked with 6-0 silk suture (Ethicon, New Brunswick, NJ, USA) and released for perfusion 60 min later. After LAD ligation, ASC-derived, low-thermogenic BAC (LBAC)-derived, or high-thermogenic BAC (HBAC)-derived enriched conditioned medium (CM) was injected at the infarct border zone for the CM-injected groups. The sham group underwent the same operation except for LAD ligation. The cardiac infarct sites were identified using subsequent 2,3,5-triphenyltetrazolium chloride (TTC, Sigma, St. Louis, MO, USA) staining. The heart tissue was chopped into four pieces, and all pieces were incubated with 1% TTC solution for 20 min at 37 °C in the dark and photographed with a digital camera.

### 2.2. RNA Sequencing and Data Analysis

Total RNA was isolated using TRIzol reagent, the RNA quality was measured with an Agilent 2100 Bioanalyzer using an RNA 6000 Nano Chip (Agilent Technologies, Amstelveen, The Netherlands), and the RNA was quantified with an ND-2000 Spectrophotometer (Thermo Fisher Scientific, Wilmington, DE, USA). The sequencing libraries were created with a SMARTer Stranded RNA-Seq Kit (TaKaRa Bio, Mountain View, CA, USA), and ribosomal RNA (rRNA) was removed with a RiboCop rRNA Depletion Kit (Lexogen, Vienna, Austria). A HiSeq 2500 system (Illumina, San Diego, CA, USA) was used for high-throughput sequencing. TopHat software [28] and ExDEGA (e-Biogen, Seoul, Republic of Korea) were utilized for mapping and data mining, respectively. The STRING v11.0 (http://string-db.org) database was used for network analysis.

### 2.3. H9c2 Cell Culture and ROS Induction

The rat cardiomyocyte line H9c2 (American Type Culture Collection; ATCC) was cultured in Dulbecco’s modified Eagle’s medium (DMEM; Gibco, Thermo Fisher Scientific, Waltham, MA, USA) with glucose (25 mM) and L-glutamine (4 mM) supplemented with 10% fetal bovine serum (FBS; Atlas Biologicals, Fort Collins, CO, USA) and 1% antibiotics (Gibco) at 37 °C under humidified conditions (5% CO_2_ atmosphere). To induce ROS production, the cells were incubated with 300 μM H_2_O_2_ (Sigma) for 3 h. After exposure to ROS, the cells were treated with ASC-, LBAC-, or HBAC-derived concentrated CM (5 μL/mL) and incubated for 2 h at 37 °C. Recombinant FGF21 (Novus Biologicals, Centennial, CO, USA) was dissolved in distilled water and the cells were incubated with different doses (10, 50, or 100 ng/mL) of FGF21.

### 2.4. Transient Nrf2 Knockdown

Target-specific siRNAs of rat *Nfe212* (Bioneer, Daejeon, Korea; #83619-1, sense (5′-3′) CUGAUCAGGCUCAGUCACU (dTdT) and antisense (5′-3′) AGUGACUGAGCCUGAUCAG (dTdT)) was used for knockdown and AccuTarget Negative Control siRNA (Bioneer) was used as a negative control. The H9c2 cells were seeded at a density of 1 × 10^5^ cells/well in a 6-well plate and then cells were transfected with siRNA (25 pmole per well) using Lipofectamine RNAiMAX (Invitrogen, Carlsbad, CA, USA) according to the manufacturer’s recommendation. After 24 h, the cells were harvested for further study.

### 2.5. Cell Viability and Cytotoxicity Assays

Cell viability was measured by the CCK-8 assay (Dogen, Seoul, Korea). Briefly, H9c2 cells were seeded at 1 × 10^4^ cells per well in a 96-well plate and treated with H_2_O_2_. After 3 h, 10 µL of CCK-8 reagent per well was added, and the cells were incubated for 1 h at 37 °C. For the cytotoxicity assay, a Lactate Dehydrogenase (LDH) Cytotoxicity Detection Kit (TaKaRa, Nojihigashi, Kusatsu, Shiga, Japan) was used. Cell culture supernatant was mixed with 100 μL of prepared LDH reagent in each well. The plate was incubated for 30 min at room temperature (RT). The optical density (OD) was measured at 450 nm with a microplate reader (Multiskan FC, Thermo Fisher Scientific).

### 2.6. Adipocyte Differentiation and CM Preparation

Rat ASCs (Cyagen Biosciences, Shanghai, China) were cultured in DMEM (Gibco) with glucose (5.56 mM), L-glutamine (4 mM), and sodium pyruvate (1 mM) supplied with 10% FBS and 1% antibiotics. For initial differentiation of the LBACs and HBACs, the medium was replaced with DMEM/F-12 (Gibco) containing 5 µg/mL insulin, 1 nM triiodothyronine (T3), 125 µM indomethacin, 2 µg/mL dexamethasone, 0.5 mM 3-isobutyl-1-methylxanthine (IBMX), and 0.5 µM rosiglitazone when the cells reached 80–90% confluence. After four days, the culture medium of the LBACs was changed to DMEM/F-12 containing 10 µg/mL insulin, whereas that of the HBACs was replaced with DMEM/F-12 including 5 µg/mL insulin, 1 nM T3, and 1 µM rosiglitazone for maturation. The cells were cultured for eight days, and the medium was replaced with new maturation medium once every two days. To prepare CM, the cells were cultured in serum-free medium for an additional 24 h. Cell debris was removed by two-step centrifugation at 480× *g* for 5 min and 2000× *g* for 10 min. The supernatant was concentrated to 1/50 using an Amicon Ultra Centrifugal Filter with a 3000 nominal molecular weight limit (EMD Millipore, Bedford, MA, USA), and the resulting CM was used for subsequent experiments.

### 2.7. Oil Red O (ORO) Staining

ORO staining was performed as described in our previous studies [27]. The ASCs and adipocytes differentiated from ASCs were fixed with 4% formaldehyde for 1 h at RT and then washed with distilled water three times. An ORO solution (Sigma) was supplied to the cells. The cells were incubated for 20 min and washed with distilled water three times. The ORO-stained cells were examined using light microscopy.

### 2.8. Adipokine Array

The secretomes of ASC-, LBAC-, and HBAC-derived CM were determined with a Rat Adipokine Array Kit (R&D Systems (ARY016), Minneapolis, MN, USA) following the manufacturer’s instructions. The intensity of each spot was quantified with ImageJ software, and the fold changes in the expression intensity in LBAC- and HBAC-derived CM were calculated relative to the intensity in ASC-derived CM.

### 2.9. Nuclear Extraction

The cytosolic and nuclear fractions were separated using NE-PER Nuclear and Cytoplasmic Extraction Reagents (Pierce; Thermo Fisher Scientific) according to the manufacturer’s instructions. In brief, the cells were washed twice using chilled phosphate-buffered saline (PBS) and centrifuged at 500 ×g for 5 min. The pellet was resuspended in 200 μL of Cytoplasmic Extraction Reagent I (CER I) and incubated for 10 min on ice. The mixture was added to 11 μL of Cytoplasmic Extraction Reagent II (CER II) and centrifuged at 16,000× *g* for 5 min again. The supernatant including the cytoplasmic extract was transferred to a new tube. The pellet fraction was resuspended in 100 μL of Nuclear Extraction Reagent (NER), mixed every 10 min for 15 s to 40 min on ice and centrifuged at 16,000× *g* to collect the nuclear extracts.

### 2.10. Western Blot Analysis

Cells were lysed with RIPA buffer (Thermo Fisher Scientific) containing 1% phosphatase inhibitor (Thermo Fisher Scientific) and 1% protease inhibitor (Santa Cruz Biotechnology, Paso Robles, CA, USA) and incubated on ice for 10 min. The supernatant was collected by centrifugation at 11,463× *g* for 7 min at 4 °C. The proteins were separated on SDS-PAGE gels and blotted onto polyvinylidene difluoride (PVDF, EMD Millipore, Burlington, MA, USA) membranes. The membranes were blocked with 5% skim milk in Tris-buffered saline with 0.1% Tween 20 (TBS-T) for 1 h and incubated with primary antibodies overnight at 4 °C. The primary antibodies were used to probe for NQO1, Catalase, SESN2, SOD1, BCL2, CIDEA, FABP4, C/EBPβ, PGC1α (1:1000; Santa Cruz Biotechnology), β-actin (1:5000; Santa Cruz Biotechnology), Caspase 9, Caspase 3, Histone H3 (1:1000; Cell Signaling, Danvers, MA, USA), NRF2 (1:500; Invitrogen), UCP-1 (1:200; Invitrogen), PPARγ (1:1000; LifeSpan BioSciences, Seattle, WA, USA), and PRDM16 (1:1000; Novus Biologicals). After washing five times with TBS-T for 5 min each time, the membranes were incubated with horseradish peroxidase (HRP)-conjugated anti-mouse IgG or anti-rabbit IgG (1:2000; Santa Cruz Biotechnology) for 1 h. After washing five times, the proteins were developed with enhanced chemiluminescence (ECL Western Blotting Detection Kit, GE Healthcare, Buckinghamshire, UK) using a ChemiDoc XRS+ machine (Bio-Rad, Hercules, CA, USA). The intensity of each band was measured with ImageJ software.

### 2.11. Quantitative Real-Time PCR (qRT-PCR) and Cell Death PathFinder RT^2^ Profiler^TM^ PCR Expression Array

Total RNA was isolated from cells using an RNA Extraction Kit (iNtRON Biotechnology, Seongnam, Korea) according to the manufacturer’s instructions. The RNA was quantified with a NanoDrop (Thermo Fisher Scientific), and reverse transcription was performed using a Maxime RT PreMix Kit (iNtRON Biotechnology). The level of each transcript was determined with a StepOnePlus Real-Time PCR system (Applied Biosystems, Foster City, CA, USA) with SYBR Green Dye (SYBR Premix Ex Taq, Tli RNase H Plus, and ROX Plus (Takara Bio, Foster City, CA, USA)). The primer sequences for qPCR are listed in Table 1. All values are displayed as the target gene expression (fold change; 2^∆∆Ct^) normalized to *Gapdh* transcript levels. The expression of 84 cell death-related genes was assessed using a Rat Cell Death PathFinder RT2 Profiler™ PCR Array (Qiagen) according to the manufacturer’s instructions. An online software program for PCR array data analysis (https://geneglobe.qiagen.com/it/analyze/, accessed on 18 September 2020, Qiagen) was used to analyze the qPCR data, and actin-beta (*Actb*), beta-2-microglobulin (*B2m*), hypoxanthine phosphoribosyl transferase 1 (*Hprt1*), lactate dehydrogenase A (*Ldha*), and ribosomal protein lateral stalk subunit P1 (*Rplp1*) were used as the reference genes. The expression of the reference genes remained constant.

### 2.12. Flow Cytometry

An Annexin-V-FITC/PI Kit (BD Biosciences, Piscataway, NJ, USA) was used to quantify apoptosis according to the manufacturer’s instructions. Briefly, harvested H9c2 cells (1 × 10^5^) were washed with chilled PBS, resuspended in binding buffer, added to 5 μL of annexin-V and propidium iodide (PI), and incubated for 15 min at RT in the dark. Cellular fluorescence was measured by flow cytometry analysis (BD AccuriC6 cytometer, BD Biosciences).

### 2.13. Immunofluorescence Staining

The H9c2 cells on 4-well slide chambers (SPL, Pocheon, Korea) were fixed with 4% formaldehyde and washed with PBS. The cells were permeabilized in PBS containing 0.2% Triton X-100 (Sigma) for 15 min, blocked in 2.5% normal horse serum (Vector Laboratories, Burlingame, CA, USA) for 1 h and stained with anti-NRF2 (1:200), anti-UCP-1 (1:200), anti-PPARγ (1:200), and anti-PRDM16 (1:200) antibodies at 4 °C overnight. After three rinses with PBS, the cells were incubated for 1 h with rhodamine-conjugated anti-rabbit IgG (1:500, Vector Laboratories) and FITC-conjugated anti-rabbit IgG (1:500, EMD Millipore) secondary antibodies. The nuclei were counterstained with DAPI (Invitrogen). MitoTracker Red (Invitrogen) was utilized for mitochondrial dyeing. All images were examined using a confocal microscope (LSM710, Carl Zeiss, Jena, Germany).

### 2.14. TUNEL Staining

Apoptosis was detected with a TUNEL Assay Kit using BrdU-Red (Abcam, Cambridge, MA, USA). Deparaffinized sections of heart tissue on slides were serially incubated with Proteinase K (20 μg/mL) for 15 min at RT, DNA Labeling Solution for 1 h at 37 °C, and 50 μL of anti-BrdU-Red antibody for 30 min at RT. PBS was used to wash the sections after all steps. Then, the slides were counterstained with Spectral DAPI and monitored using an LSM700 confocal laser scanning microscope (Carl Zeiss, Oberkochen, Germany).

### 2.15. Immunohistochemical Staining

Paraffin-embedded heart tissues were sectioned (4 µm), deparaffinized, and rehydrated. The slides were incubated with 3% hydrogen peroxide to neutralize endogenous peroxidases and blocked in 2.5% normal horse serum. A rabbit anti-NRF2 antibody (1:200) was used as the primary antibody at 4 °C overnight in a humid chamber, and a rhodamine-labeled secondary antibody was subsequently used. The nuclei were stained with DAPI.

### 2.16. Heart Function Assessment

For invasive hemodynamics, left ventricular catheterization was carried out three weeks after surgery. A Millar pressure-volume catheter (SPR-838, Millar Instruments, Inc., Houston, TX, USA) was placed in the left ventricular cavity via the right carotid artery. After stabilization for 5 min, ventricular pressure and volume loops were measured in real-time, and all results were analyzed with LabChart v8.1.5 software (Millar).

### 2.17. Statistical Analysis

Comparative analysis was performed by one-way analysis of variance (ANOVA) with the Statistical Package of Social Science (SPSS, version 17) program. All results are displayed as the mean ± SEM. Significant differences between groups were measured using a protected least-significant difference (LSD) test. A *p* value less than 0.05 was considered to indicate statistical significance.

## 3. Results

### 3.1. Transcriptome Analysis of Myocardial IR Injury in Rat Hearts

**Figure 1:**
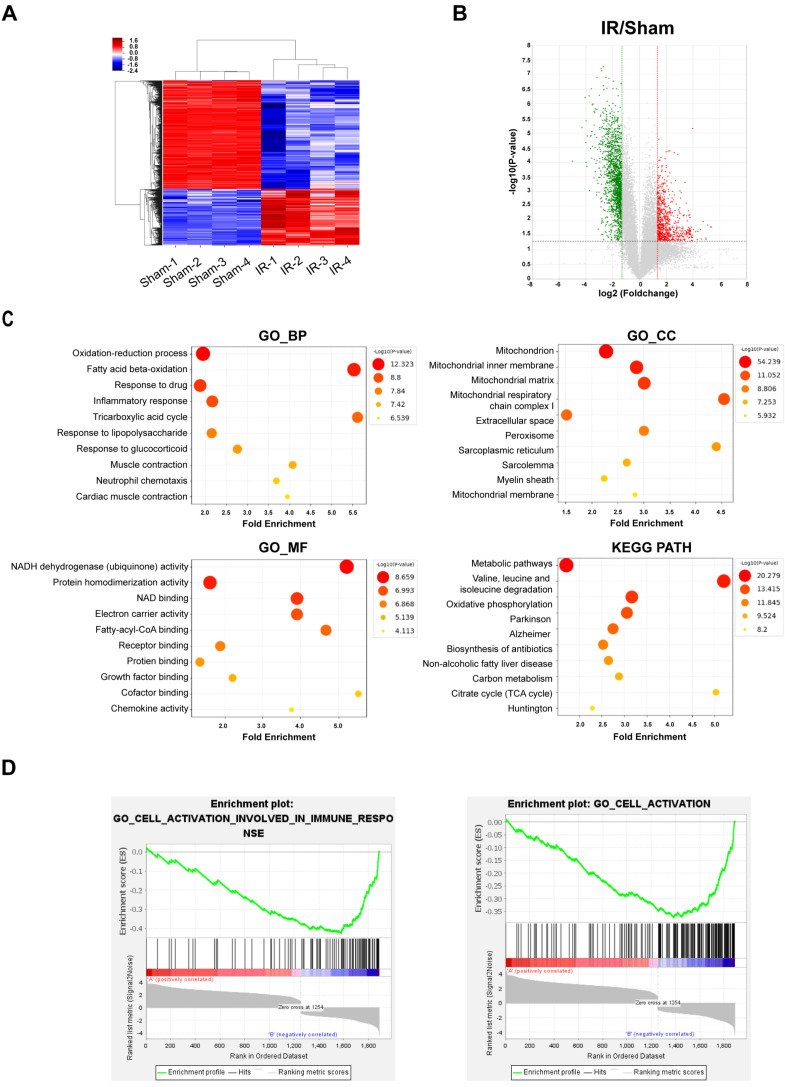
Identification of differentially expressed genes (DEGs) by transcriptome sequencing and functional analysis in IR rat hearts. (**A**) Hierarchical cluster analysis of RNA sequencing data. (**B**) Volcano plot depicting the differential RNAs, with the vertical lines corresponding to 2-fold up- and downregulation and the horizontal line representing a *p* value of 0.05. (**C**) GO analysis of differentially expressed RNAs. (**D**) GSEA enrichment plots for two hallmark gene sets enriched in IR rat hearts.

To identify differentially expressed transcriptomes between sham and IR rat hearts, we performed total RNA-Seq using HiSeq 2500. The results showed that a total of 6486 genes were differentially expressed with a >2.5-fold change, with *p* < 0.05. A hierarchical cluster was used to show the different RNA profiles, and a volcano plot was used to provide an overview of the differentially expressed RNAs between the two groups (Figure 1A,B). Gene Ontology (GO) enrichment analysis was used to show the functional classifications for differentially expressed genes, and the Kyoto Encyclopedia of Genes and Genomes (KEGG) pathway enrichment identified the relevant signaling pathways of these genes. The top 10 most enriched GO terms in the biological process (BP), cellular component (CC), and molecular function (MF) categories and the KEGG pathways are shown in Figure 1C. In addition, we conducted gene set enrichment analysis (GSEA) with the differentially expressed genes to identify the gene set hallmarks. The results showed that there was strong enrichment in IR rat hearts for the “Cell activation involved in immune response” and “Cell activation” terms (Figure 1D and Figure S1).

### 3.2. HBAC Differentiation for IR Rat Heart Therapy

**Figure 2:**
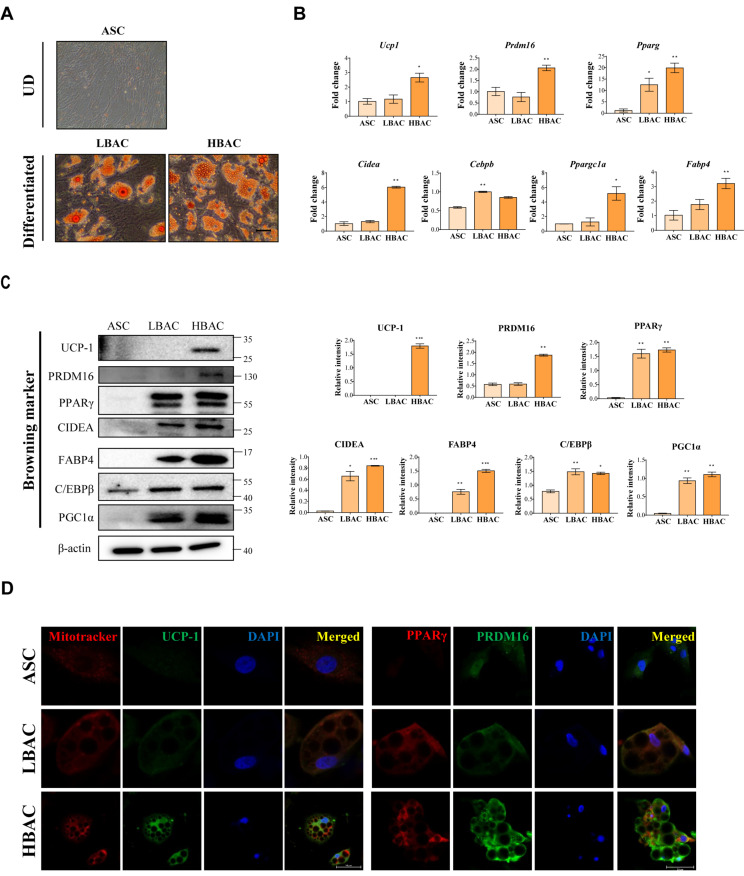
Beige adipocyte differentiation of rat adipose-derived stem cells. (**A**) Representative microscopic images of adipocytes stained with ORO (magnification, ×100, Scale bar = 100 μm). (**B**) Assessment of the mRNA expression of browning markers by qPCR. (**C**) Validation of differentially regulated browning markers by immunoblot analysis. The intensity of each band was quantified with ImageJ software. The data are shown as the mean ± standard error of the mean (*n* = 3). * *p* < 0.05, ** *p* < 0.01, and *** *p* < 0.001 vs. ASC. (**D**) Assessment of the expression of browning markers by immunofluorescence staining. Scale bar = 50 μm. UD means “undifferentiated”.

Thermogenic brown/beige adipocytes have potential utility for the development of therapeutics to treat metabolic diseases, but their potential utility for cell therapy has been less explored. Here, we first differentiated LBACs and HBACs from rat adipose-derived stem cells for IR rat heart treatment (Figure 2). HBACs exhibited high lipid drop formation and elevated levels of thermogenic markers compared with ASCs and LBACs. qPCR revealed the induction of brown/beige adipocyte-specific genes including *Ucp1*, *Prdm16*, *Pparg*, *Cidea*, *Cebpb*, *Ppargc1a*, and *Fabp4* (Figure 2B). We observed higher expression of genes in HBACs than in ASCs/LBACs. Immunoblot analysis also showed that BAC-specific markers were more highly expressed in HBACs than in ASCs/LBACs (Figure 2C). Immunostaining of cells exhibited clusters of lipid droplets surrounded by an abundance of mitochondria and elevated expression of UCP-1, PPARγ, and PRDM16 in HBACs (Figure 2D).

### 3.3. Effects of Beige Adipocyte Secretions on Cardiomyocytes under Oxidative Stress

Oxidative stress is one of the most important pathological mechanisms in reperfusion injury and causes cardiomyocyte damage and cardiac dysfunction. In this study, to determine the effects of beige adipocyte secretions on cardiomyocytes under oxidative stress, we treated H9c2 cardiomyocytes with H_2_O_2_ and then exposed them to CM for 1 h.

**Figure 3:**
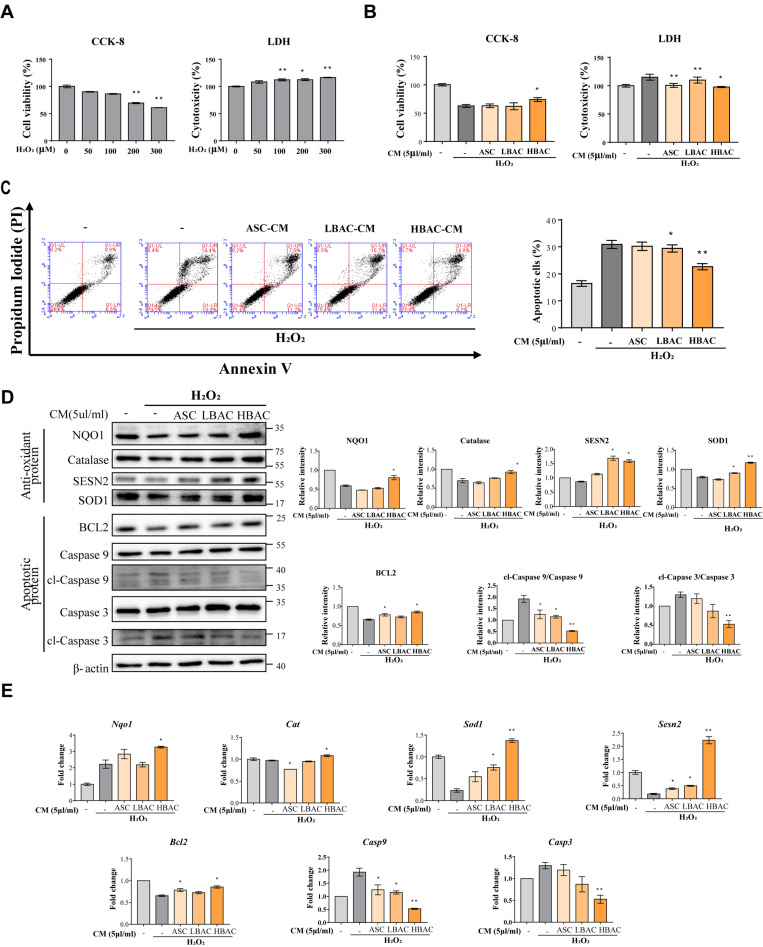
Regulation of antioxidant and apoptotic factors by beige adipocyte secretions. (**A**) Cell viability and cytotoxicity in H9c2 cells under H_2_O_2_ stimulation. (**B**) Cell viability and cytotoxicity in H9c2 cells under H_2_O_2_ stimulation with CM. H9c2 cells were treated with 300 μM H_2_O_2_ for 3 h and then exposed to CM for 1 h. The data are shown as the mean ± standard error of the mean (*n* = 5). (**C**) Annexin V/PI staining as detected by flow cytometry. (**D**,**E**) Differential expression of antioxidant/apoptotic factors in H9c2 cells by ASC, LBAC, or HBAC-derived CM treatment. The factors were determined using western blot analysis (**D**) and qPCR (**E**). The data are shown as the mean ± standard error of the mean (*n* = 3). * *p* < 0.05 and ** *p* < 0.01 vs. non-treated (**A**) or H_2_O_2_-treated group (**B**–**E**). cl means ”cleaved form”.

H9c2 cell viability decreased in response to H_2_O_2_ stimulation, and the cytotoxicity increased in a H_2_O_2_ concentration-dependent manner (Figure 3A). However, HBAC-CM treatment increased the viability of H9c2 cells stimulated with H_2_O_2_ and decreased the cytotoxicity of H_2_O_2_ toward cardiomyocytes (Figure 3B). Based on this result, we used a concentration on 300 μM of H_2_O_2_ in subsequent experiments. To determine whether the CM affected cell viability, we examined apoptotic cell death by flow cytometry analysis with Annexin V-PI staining. As expected, HBAC-CM treatment enhanced cardiomyocyte viability, reducing oxidative stress-induced apoptotic cell death (Figure 3C). To evaluate the relative contributions of antioxidative and antiapoptotic effects, we observed the expression levels of antioxidant proteins and apoptotic proteins after the treatment of thermogenic beige adipocyte with CM (Figure 3D). In addition, it was confirmed at the mRNA level by qPCR (Figure 3E). Antioxidant protein (NQO-1, Catalase, SESN2, SOD1) expression was higher in the HBAC-CM group than in the other groups (ASC-CM and LBAC-CM), while apoptotic protein (Caspase 3 and Caspase 9) expression levels were decreased in HBAC-CM-treated cells.

### 3.4. Regulation of Multiple Cell Death Signals by Beige Adipocyte Secretions in Cardiomyocytes under Oxidative Stress

**Figure 4:**
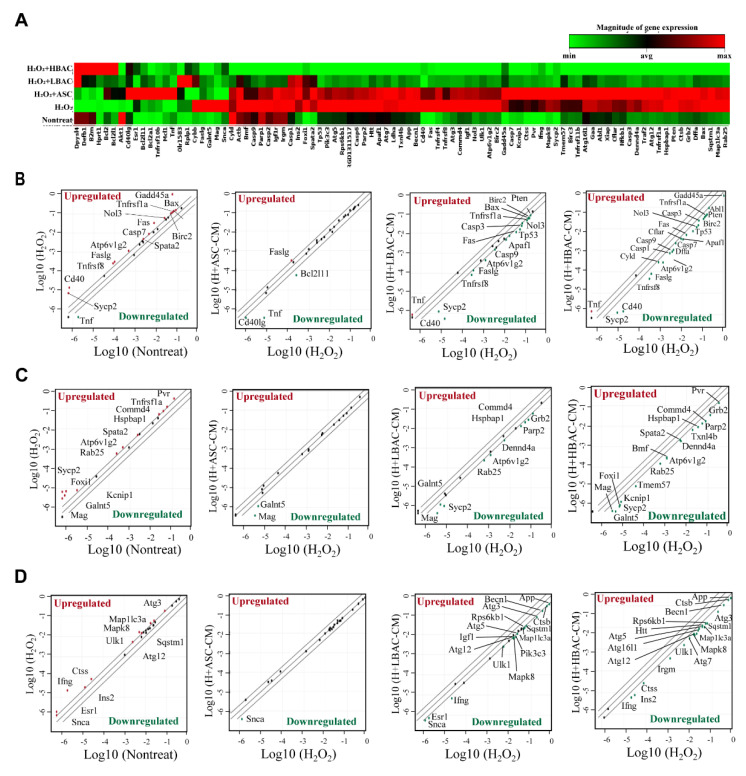
Regulation of multiple cell death signals by beige adipocyte secretions under oxidative stress. (**A**) Hierarchical cluster map of gene expression determined by PCR array. (**B**) Apoptosis signals. (**C**) Necroptosis signals. (**D**) Autophagy signals. The relative expression levels of each gene are plotted for each group comparison (nontreated vs. H_2_O_2_-stimulated sample, H_2_O_2_-stimulated sample vs. CM-treated sample). The diagonals show the same representation in both groups with a 2-fold change boundary. Upregulated genes with greater than 2-fold changes are marked above the middle line, and downregulated genes are marked below the line in each graph.

To investigate the involvement of cell death signals in oxidative stress induced in cardiomyocytes, we evaluated the expression of multiple cell death signal-associated genes in CM-treated H9c2 cells compared with H_2_O_2_-stimulated H9c2 cells using a Cell Death PathFinder RT^2^ Profiler PCR array. This array profiles the expression of 84 key genes representative of cell death signals involved in apoptosis, necroptosis, and autophagy. The heatmap showed that most of the cell death-related genes were upregulated with H_2_O_2_ stimulation in H9c2 cells (Figure 4A). Surprisingly, multiple cell death signal-related genes were significantly downregulated after HBAC treatment in H_2_O_2_-stimulated H9c2 cells. The relative expression levels of each gene in two samples (nontreated vs. H_2_O_2_-stimulated sample, H_2_O_2_-stimulated sample vs. CM-treated sample) were plotted against each other in the scatter plot, which revealed the up- and downregulated genes in the experimental samples (Figure 4B–D). Oxidative stress in cardiomyocytes influenced the expression of cell death genes involved mostly in apoptosis, necroptosis, and autophagy signals. The expression of apoptotic factors such as *Cd40*, *Gadd45a*, and *Sycp2* was most strongly upregulated by H_2_O_2_ stimulation in H9c2 cells. In addition, 12 out of 27 proapoptotic genes were upregulated with more than 2-fold gene expression changes after H_2_O_2_ stimulation, while the expression of the three genes *Bcl2l11*, *Cd40g*, and *Tnf* was unaffected (Figure 4B). Additionally, upregulation of 12 out of 22 necrosis-related genes was detected after H_2_O_2_ stimulation in H9c2 cells, while the expression of necrotic genes was significantly downregulated in HBAC-CM-treated cells (Figure 4C). Moreover, 11 of 31 autophagy-associated genes were upregulated in H_2_O_2_-stimulated H9c2 cells, while the expressed genes were downregulated after HBAC-CM treatment (Figure 4D).

### 3.5. Identification of Adipocyte Secretion Factors and Regulation of Cardiomyocytes after Injury

**Figure 5:**
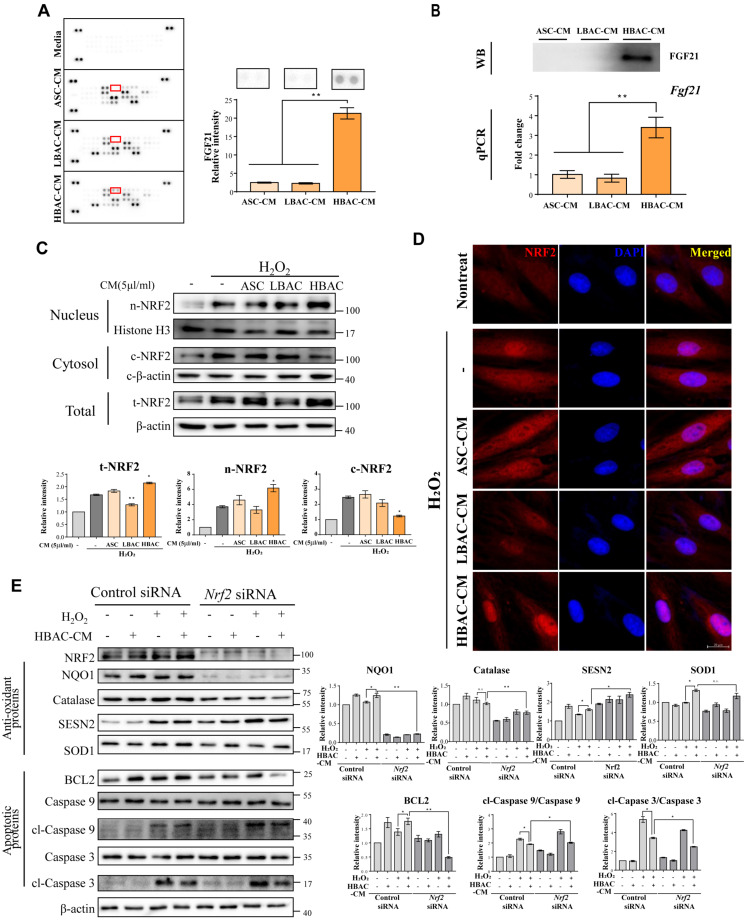
Brite adipocyte FGF21 attenuates cardiac IR injury by modulating NRF2. (**A**) Elevated FGF21 secretion from beige adipocytes in the adipokine array. (**B**) Validation of FGF21 protein expression in ASC, LBAC, or HBAC-derived CM by western blot analysis (up) and FGF21 gene expression in ASC, LBAC, or HBAC using qPCR (down). (**C**) Oxidative stress is alleviated by NRF2 translocation. * *p* < 0.05 and ** *p* < 0.01 vs. H_2_O_2_-treated group. (**D**) Representative images of NRF2 immunofluorescence and DAPI staining. Scale bar = 200 μm. (**E**) Effect of *Nrf2* knockdown on the expression of oxidant and apoptosis-related proteins. The data are shown as the mean ± standard error of the mean (*n* = 3). * *p* < 0.05, ** *p* < 0.01. cl means “cleaved form”.

To study which brown adipokine plays a role in metabolic crosstalk and ischemic heart repair, we analyzed the CM of each cell culture using an adipokine array. We found elevated levels of FGF21 in HBAC-CM by both the adipokine array and western blot analysis (Figure 5A,B). We also confirmed that the mRNA level of FGF21 was increased in HBAC (Figure 5B (down)). NRF2 is a key molecule inducing endogenous antioxidant and anti-inflammatory responses to oxidative stress. FGF21 probably protects cardiomyocytes from oxidative stress via its antioxidative function. We found enhanced NRF2 expression levels in HBAC-CM-treated cardiomyocytes under oxidative stress by both western blot and immunohistochemistry (Figure 5C,D). Antioxidant and antiapoptotic factors were upregulated by high levels of secreted FGF21 after HBAC-CM treatment in H9c2 cardiomyocytes, whereas FGF21 in HBAC-CM did not affect antioxidative or antiapoptotic cell death in H9c2 cardiomyocytes with *Nrf2* knockdown (Figure 5E).

### 3.6. Effect of Treatment with the Beige Adipocyte Secretion Factor FGF21 on Cardiomyocytes under Oxidative Stress

**Figure 6:**
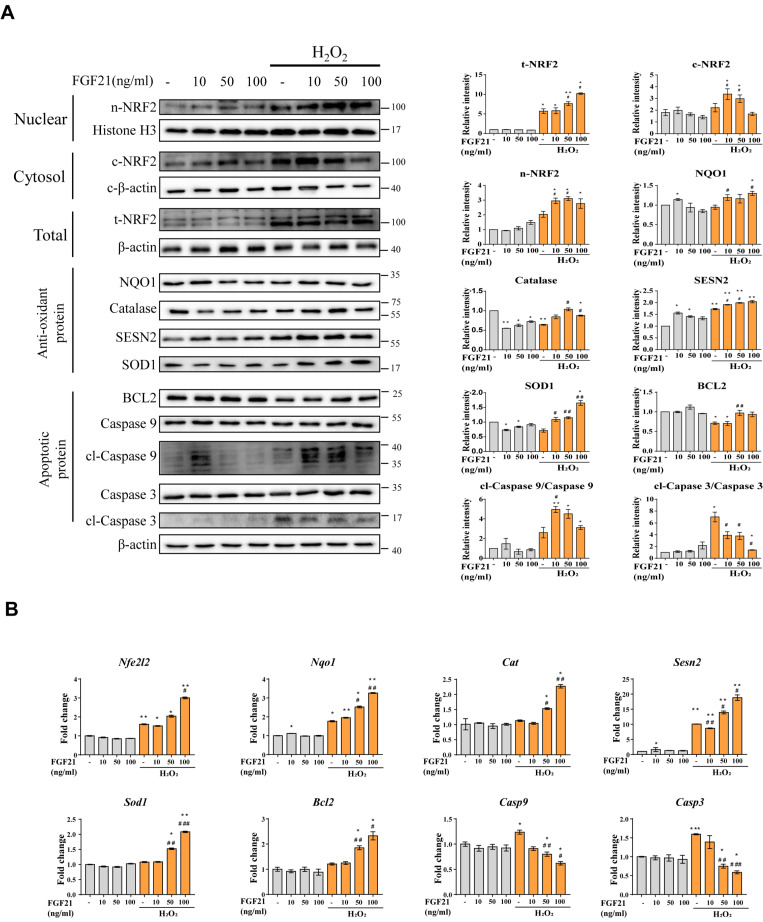
Effect of FGF21 treatment on H_2_O_2_-induced oxidative stress and apoptosis in H9c2 cells. Assessment of the differential expressed antioxidant/apoptotic factors by western blot (**A**) and qPCR (**B**). The data are shown as the mean ± standard error of the mean (*n* = 3). * *p* < 0.05 and ** *p* < 0.01 vs. non-treated group; *# p* < 0.05, *## p* < 0.01 and *### p* < 0.001 vs. H_2_O_2_-treated group. cl means “cleaved form”.

We investigated whether FGF21 confers beneficial effects on antioxidant and antiapoptotic factors in oxidatively stressed cardiomyocytes (Figure 6). The expression levels of antioxidative factors were upregulated according to FGF21 concentration, with enhanced NRF2 expression in H9c2 cardiomyocytes under H_2_O_2_-induced oxidative stress (Figure 6A). In contrast, apoptotic factors were downregulated with FGF21 treatment in oxidatively stressed cardiomyocytes (Figure 6B).

### 3.7. Effect of Beige Adipocyte Secretions on IR Rat Hearts

**Figure 7:**
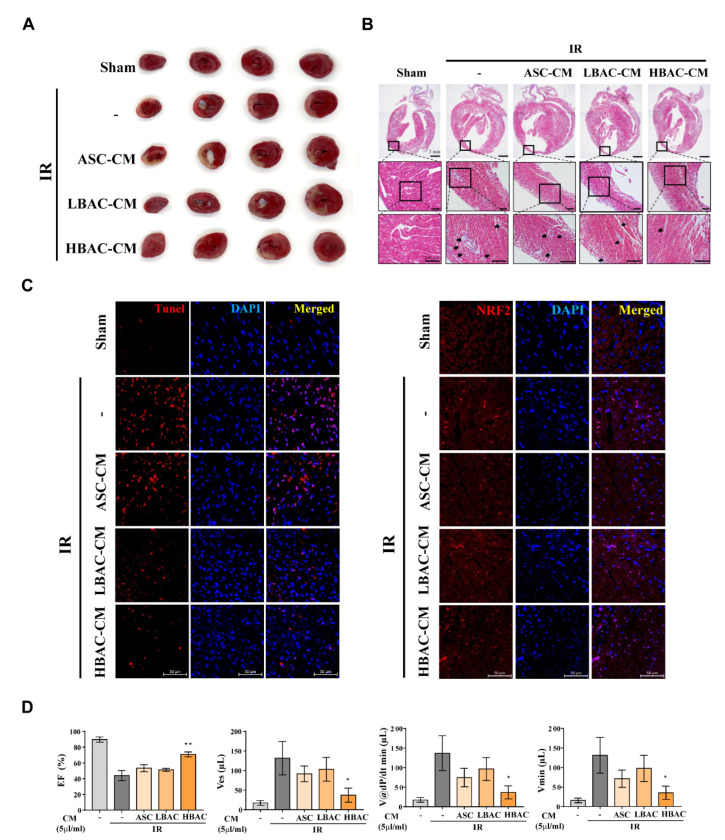
Effect of beige adipocyte secretions on IR rat hearts. (**A**) Triphenyl tetrazolium chloride (TTC) staining showing the infarct areas in transverse sections. Representative images of rat heart slices stained with TTC. (**B**) Histological analysis of IR rat hearts after beige adipocyte secretion injection. Cardiac fibrosis was evaluated by Masson’s trichrome staining. Scale bar = 2 mm (upper panel images), 200 μm (down panel images). (**C**) Representative images of TUNEL staining and NRF2 immunofluorescence. Scale bar = 50 μm. (**D**) Cardiac function was valuated with a Millar catheter in rat heart at three weeks after IR and treatment of CMs. EF, ejection fraction; ESV, end-systolic volume; V@dP/dt min, volume at dP/dt min. The data are shown as the mean ± standard error of the mean (*n* = 5). * *p* < 0.05 and ** *p* < 0.01 vs. IR group.

Since beige adipocyte secretions (including the secreted factor FGF21) ameliorated oxidative and apoptotic damage in cardiomyocytes under oxidative stress, we next investigated whether beige adipocyte secretions alleviated IR rat heart injury (Figure 7). We determined the functional role of beige adipocyte secretions in ischemic heart injury and found that the secretions significantly reduced the size of the ischemic area in IR rat hearts (Figure 7A). Histochemical staining of the heart also demonstrated that beige adipocyte secretions significantly attenuated ischemic heart tissue injury (Figure 7B). TUNEL and IR staining analyses showed that the cardiomyocyte cell death induced by IR was markedly reduced in HBAC-CM-treated rat hearts (Figure 7C). IR rat heart tissue showed significantly increased cardiomyocyte cell death, but treatment with HBAC-CM drastically decreased this ischemic cell death, causing greater nuclear translocation of NRF2 than that observed in IR rat hearts.

In addition, beige adipocyte secretions significantly improved cardiac function parameters including the ejection fraction (EF), end-systolic volume (ESV), and volume at dP/dt min (V@dP/dt min) from the levels in IR rat hearts (Figure 7D). Altogether, the findings indicate that beige adipocyte secretions suppress cell death pathways and cooperatively inhibit IR-induced cardiomyocyte cell death in rat hearts.

## 4. Discussion

In the present study, we found that treatment with HBAC-CM (including high levels of secreted FGF21) enhanced the expression of antioxidant and antiapoptotic factors in oxidative stress-induced H9c2 cardiomyocytes. In contrast, HBAC-CM did not affect antioxidative or antiapoptotic cell death in H9c2 cardiomyocytes with *Nrf2* knockdown. These results show that FGF21 mediates antioxidative and antiapoptotic effects through NRF2 activation. However, HBAC-CM may include FGF21 as well as other factors that have an antioxidative and antiapoptotic effect. Mechanistically, we found that FGF21 in HBAC-CM induced NRF2 translocation into the nucleus and alleviated oxidative stress-induced cardiomyocyte apoptosis. Moreover, we obtained direct evidence that the antioxidant and antiapoptotic effects occurred through NRF2 induction by FGF21 in the context of H_2_O_2_-induced H9c2 cardiomyocyte injury. Based on these findings, we propose that HBAC-CM is a potential therapeutic agent against oxidative stress-induced cardiac injury (Figure 5 and Figure 6). Besides oxidative stress and apoptosis, inflammation is a major driver of myocardial I/R injury [29]. Activation of FGF21/NRF2 has been identified as an antioxidant effect induced by AMPK not only in cell apoptosis, but also in inflammation [30]. Therefore, the protective effect of FGF21 in IR injury may be related to inflammation in this study. However, the cardioprotective effect of HBAC-CM in in vivo study cannot be concluded as the solo effect of cardiomyocytes. This is because FGF21 induces antiapoptotic and/or anti-inflammatory effects in other cells such as endothelial cells and leukocytes of myocardium [31,32]

In a recent study, both endogenous and exogenous FGF21 prevented cardiac apoptosis in type 1 diabetes mellitus (T1DM) mice by inhibiting lipotoxicity [30]. Furthermore, it has been reported that FGF21 prevents diabetic cardiomyopathy via AMPK-mediated antioxidation and lipid-lowering effects in the heart. FNDC5/irisin, a cold-induced endocrine activator of brown fat, alleviates oxidative stress and cardiomyocyte apoptosis in doxorubicin (DOX)-induced cardiotoxicity by activating AKT [33]. Based on these findings, it has been hypothesized that FNDC5/irisin is a potential therapeutic agent against DOX-induced cardiotoxicity. Although AT-derived adipokines are known to play roles in the regulation of cardiovascular function, these typical adipokines are expressed at low levels in BAT. Recently, batokines, which act in a paracrine or autocrine manner in adipocytes, have been identified in BAT [34]. FGF21, one of the first identified batokines, promotes glucose utilization, improves glycemia and lipidemia, and induces adipocyte browning [35,36]. Cold-induced thermogenic activation or pharmacological activation of BAT can promote FGF21 production in BACs [37]. Notably, excess visceral fat located adjacent to the heart and coronary arteries is associated with increased cardiovascular risk, and dysfunctional AT in pathophysiological conditions regulates vascular function and secretes factors that induce atherogenesis [38]. Conversely, brown and beige ATs utilize glucose and lipids to generate heat and are associated with improved cardiometabolic health. Cardiac and thoracic perivascular adipose tissues are now understood to be composed of BAT in the healthy state and to undergo a brown-to-white transition during conditions such as obesity, which may be a driving factor of cardiovascular disease [39].

The transcription factor NRF2 is a key modulator that preserves redox balance and regulates the genes that encode antioxidant proteins [40]. Recently, increasing evidence has implicated the activation of the NRF2 pathway in alleviating the progression of ischemic heart diseases including myocardial infarction [41,42,43]. Here, HBAC-CM treatment enhanced the activation of NRF2 in cardiomyocytes from IR-injured hearts upon oxidative stress. Thus, we further investigated the involvement of NRF2 activation in cardioprotection of IR-injured hearts by HBAC-CM treatment. Notably, *Nrf2* knockdown blunted the protective efficacy of HBAC-CM treatment against cardiomyocyte apoptosis and oxidative stress in H9c2 cardiomyocytes.

Importantly, compelling evidence suggests that activation of NRF2 can reduce myocardial infarct size and promote the recovery of cardiac function by suppressing cell injury and the oxidative stress response during myocardial IR. Therefore, the current findings indicate the involvement of NRF2 activation in cardioprotection mediated by HBAC-CM treatment in H9c2 cardiomyocytes with induced oxidative stress. Bidirectional crosstalk between adipose tissue and myocardium is important for maintaining the normal functioning of both organs. Therefore, in this study, we first investigated brown adipocyte secretion factors involved in the regulation of antioxidant and anti-apoptotic responses in IR rat hearts. Moreover, we propose that brown adipocyte-secreting factor may constitute a novel therapeutic strategy for cardioprotection in ischemic heart disease.

Interestingly, some previous studies have shown that thermogenesis is not uniformly activated in all BACs [44]. For example, BACs have been shown to have heterogeneous expression of UCP1. In addition, in vitro-cultured BACs show heterogeneous mitochondrial membrane potentials. However, the thermogenic and metabolic heterogeneity of BACs within the same BAT in vivo remains largely uncharacterized. A recent study utilized single-cell RNA sequencing and 3D tissue profiling as well as multiple mouse models to genetically label different cell populations in brown fat with low thermogenic activity coexisting with classical HBACs within BAT [45]. Compared with HBACs, these LBACs had substantially lower *Ucp1* and *Adipoq* expression, larger lipid droplets, and lower mitochondrial content. Functional analysis showed that, compared with HBACs, LBACs have significantly lower basal mitochondrial respiration and are specialized for fatty acid uptake. In our study, rat adipose-derived stem cells were differentiated into LBACs and HBACs to study the different effects on cardioprotection caused by the heterogeneous expression of brown adipocytes.

The use of animal models for the study of cardiac I/R is widely adopted, but rodent models of myocardial I/R do not completely recapitulate human I/R for several reasons. First, unlike humans, animals do not have comorbidities that might directly or indirectly impact I/R as well as responses to treatments. Second, tissue destruction and inflammation by surgical incision can affect the MI effect. Third, commonly adopted medications such as anesthetics, P2Y12 inhibitors, and statins may already exert cardioprotective effects, which may dilute the effects of the proposed cardioprotective intervention when reproduced in clinically relevant scenarios in animals or in clinical trials. Therefore, various approaches to overcome these limitations have been attempted to this day [46].

## 5. Conclusions

Our present study found enhanced NRF2 expression levels in HBAC-CM-treated cardiomyocytes under oxidative stress than in LBAC-CM-treated cardiomyocytes. In addition, the expression of antioxidant and antiapoptotic genes was upregulated by highly secreted FGF21 after HBAC-CM treatment compared to LBAC-CM treatment in H9c2 cardiomyocytes via NRF2 activation.

## Figures and Tables

**Table 1:** Primer sequences used for quantitative real-time PCR.

Gene	Forward Sequence	Reverse Sequence
*Ucp1*	ACAAATAGCCCTGGTGGCTG	AACTCACCATCTTGGCTCGG
*Prdm16*	CCTAACAACGTGCTCAGGGT	CTCTCTGCACGAAGTCAGCA
*Pparg*	GGAGATCTCCAGTGATATCGACCA	ACGGCTTCTACGGATCGAAACT
*Cidea*	GACCCAGCTTTCACGCAAT	TCTGCCTCCTTTCTCTTGCG
*Cebpb*	AATCCGGATCAAACGTGGCT	CCCCGCAGGAACATCTTTAAGT
*Pargc1a*	TTCAGGAGCTGGATGGCTTG	AGATCTGGGCAAAGAGGCTG
*Fabp4*	GTCCTGGTACATGTGCAGAA	CTCTTGTAGAAGTCACGCCT
*Fgf21*	TCCAGTTTGGGGGTCAAGTC	GACTTTCTGGACTGCGGTGT
*Nfe2l2*	TTTGTAGATGACCATGAGTCGC	ATGTCCTGCTGTATGCTGCTT
*Nqo1*	AGCCCTGATTGTATTGGCCC	GATTCGACCACCTCCCATCC
*Cat*	GCTCCGCAATCCTACACCAT	GTGGTCAGGACATCGGGTTT
*Sens2*	TACCTTAGCAGCTTCTGGCG	AGGTAAGAACACTGGTGGCG
*Sod1*	TTTTGCTCTCCCAGGTTCCG	TCGAAGTGAATGACGCCCTG
*Sod2*	ACCGAGGAGAAGTACCACGA	TGGGTTCTCCACCACCCTTA
*Hmax1*	ACATGGCCTTCTGGTATGGG	ATGAGTACCTCCCACCTCGT
*Gapdh*	AGTGCCAGCCTCGTCTCATA	GAAGGGGTCGTTGATGGCAA

## Data Availability

Not applicable.

## Supplementary Materials

The following supporting information can be downloaded at: https://www.mdpi.com/article/10.3390/cells11030567/s1, Figure S1. Heatmap of gene list for highlighted phenotype in gene set enrichment analysis.
