# Supplementary figures and images for: Brite Adipocyte FGF21 Attenuates Cardiac Ischemia/Reperfusion Injury in Rat Hearts by Modulating NRF2
Source: Cells. 2022 Feb 6;11(3):567. doi: 10.3390/cells11030567 (PMC8833946; doi:10.3390/cells11030567)

**Figure S1.** Heatmap of gene list for highlighted phenotype in gene set enrichment analysis

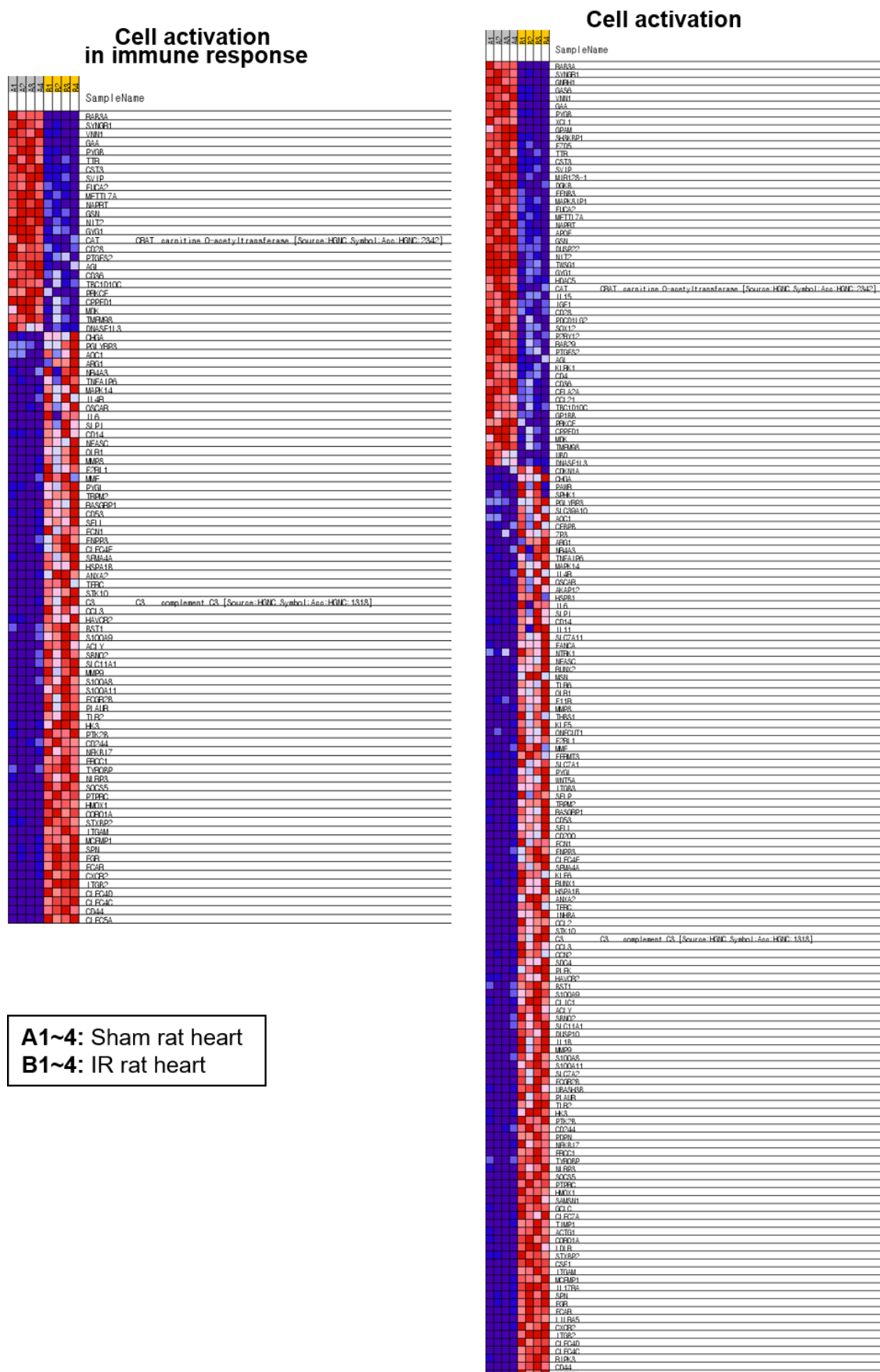

Supplement: Supplementary file 1 [file cells-11-00567-s001.zip › cells-1536070-supplementary.pdf]
